# Supplementary material for: Understanding others’ distal goals from proximal communicative actions
Source: PLoS One. 2023 Jan 20;18(1):e0280265. doi: 10.1371/journal.pone.0280265 (PMC9858010; doi:10.1371/journal.pone.0280265)
Supplement: S1 Appendix — A detailed description of the procedure used to record, exaggerate and rescale the movements. (DOCX) [file pone.0280265.s001.docx]

**Supplementary material 1**

**Movement recording and exaggeration**

Spontaneous goal-directed movements were collected by the first author using an in-house PsychoPy script that recorded mouse movements continuously within a setup that looked identical to the layout of the Proximal goal condition in Experiment 1. The animated box was attached to the mouse cursor. Cursor movements were constrained by locking them to the horizontal axis, resulting in one-dimensional sliding movements. Additionally, the animated box could only move in one direction, from the left side of the screen towards the green targets on the right.

To avoid any biases in the collection of these movements, our in-house script was set to randomly select trials to near and far targets, until a nearly equal number of at least 50 near and 50 far movements were recorded. This procedure led to a total of 105 movements (see Fig 1). We then smoothened each individual movement and averaged all near and all far movements, respectively, thus obtaining two non-exaggerated movements, one for each target location (henceforth “Normal near” and “Normal far” movements). This averaging procedure was key, as it allowed us to identify systematic differences between near and far movements (e.g., peak velocity), while also controlling for more subtle differences between individual movements (e.g., in jitter). Importantly, the averaging procedure preserved distinguishable human-like features (e.g., bell-shaped velocity profile, with a faster initial phase and slower final phase) which have been frequently reported in studies looking at rapid aiming movements (e.g., Jeannerod, 1984)

**Fig 1**. **Unsmoothed velocity profiles of sliding movements**

Subset of raw velocity profiles of sliding movements recorded, before averaging and smoothening. Movements directed to the near target are colored, while those directed to the far target are colored in blue.

Exaggerated movements were generated in three steps. First, we computed the standard deviation of near and far movements separately. Then, we identified the highest value (peak velocity) for each averaged (i.e. Normal near and Normal far) movement. Finally, we rescaled both of these movements by either subtracting one and two standard deviations from the peak velocity of the Normal near movement, or by adding one and two standard deviations to the peak velocity of the Normal far movement.

**Movement rescaling in Distal goal condition**

For the Distal goal condition the six Normal and Exaggerated movements were reshaped so that their endpoints would all converge towards the middle of the screen. To do so, we used the “rescale” function in R Studio to manually specify the maximum values of the location vectors of each movement.

The rescaling procedure yielded velocity profiles that, unsurprisingly, differed in average velocity from the original velocity profiles in the Proximal goal condition. This is simply due to the fact that the change of the movement endpoints via rescaling implies that the new velocity profiles will be either compressed (when the endpoint is shifted closer to the movement origin) or expanded (when the endpoint is shifted away from the movement origin). As a consequence of this, the difference in peak velocity between the two non-exaggerated (i.e. Normal) movements in this condition was inverted with respect to the original movements in the Proximal goal condition. Specifically, while in the Proximal goal condition the Normal movement directed towards the far target (i.e. Normal far) had a higher peak velocity than the one directed towards the near target (i.e. Normal near), this relationship was reversed in the Distal goal condition, where the Normal movement directed to the near target (i.e. Normal near) became slightly faster than the one directed to the far target (i.e. Normal far). Since we hypothesized that participants would try to produce consistent mappings based on the velocity of the movements, we decided to account for this specific reversal in the velocity of the two Normal movements in our analyses of the Distal goal condition.

**Data preparation in Distal goal condition**

As we pointed out above, the rescaling procedure led to a reversal in the velocity profiles of the two Normal movements in the Distal goal condition, such that the originally faster movement (i.e. Normal far) became slightly slower than the originally slower one (i.e. Normal near). To account for this reversal in our analyses, we decided to code “f” key responses for Normal near movements as Iconic, and “n” key responses for Normal far movements as Non-iconic in this condition. We applied the same conversion with the Normal far movements (i.e. “n” key responses for Normal far movements were coded as Iconic, while “f” key responses for Normal far movements were coded as Non-iconic). This is in line with our hypothesis that faster movements will be more likely to be mapped to far target locations, while slower ones will be mapped to near target locations (Jeannerod, 1984).

**References**

Jeannerod, M. (1984) The Timing of Natural Prehension Movements, *Journal of Motor Behavior*, 16:3, 235-254.
