# Supplementary material for: Understanding others’ distal goals from proximal communicative actions
Source: PLoS One. 2023 Jan 20;18(1):e0280265. doi: 10.1371/journal.pone.0280265 (PMC9858010; doi:10.1371/journal.pone.0280265)
Supplement: S2 Appendix — Description and analyses of a control condition in Experiment 1. (DOCX) [file pone.0280265.s002.docx]

**Supplementary material 2**

**Control condition - Partial distal goal**

We added a third, control condition where participants were presented with movement animations in which the proximal and distal goals were separated, but this separation was not made explicit to participants. This condition was similar to the Proximal goal condition in that the sliding movement looked as if it was achieving a proximal goal directly. However, and unbeknownst to participants, the sliding movement ended in between the two final locations and thus did not actually fulfil the distal goal (Fig 1 and 2).

**Fig 1. Experimental layout used in the Partial distal goal condition**.

The black dotted line represents the outline of the occluded area during trials, where the (near and far) final locations are displayed in light green. The red hexagon indicates the movement endpoint. This red figure was not shown to participants during the experiment but is used for illustration purposes only.

**Fig 2 Velocity profiles in Partial distal goal condition.**

Normal movements are colored in green, Exaggerated movements in blue, and Very Exaggerated ones in yellow. The black dotted line represents the outline of the occluded area during trials.

Unlike the Distal goal condition, where participants could directly see the separation between the proximal and distal goal because the movement endpoints converged towards a visible area in the middle of the screen, the Partial distal goal condition was introduced as a control to assess how participants would interpret reshaped movement animations that converged towards a single, although this time occluded, endpoint. Importantly, the fact that the movements were converging towards the middle of the two target locations was never revealed to participants, who were led to believe that the box was being placed within one of the green targets during familiarization. Consequently, the Partial distal goal condition allowed us to examine closely the effects of the rescaling procedure on participant’s answers, and isolate these effects from the ones introduced in Distal goal by a visible separation between proximal and distal goals.

**Results**

*Data preparation* As in the Distal goal condition, we accounted for the reversal between the two non-exaggerated movement by coding “f” key responses for Normal near movements and “n” key responses for Normal far movements as Iconic. “n” key responses for Normal near movements and “f” key responses for Normal far movements were therefore coded as Non-iconic.

**Mapping consistency** Using Bonferroni-corrected one-sample t-tests we compared the Consistency scores for each degree of exaggeration to 0 (Fig 3A) The test revealed significant differences in Consistency scores across all degrees of exaggeration (all *t*(24) > 2.2, *p* < .001, *d* > 0.6). When comparing these scores across the three degrees of exaggeration, we found, as expected, significant differences between Normal and both exaggerated movements (Exaggerated: *t*(142) = 6.5, *p* < .001, *d* = 1.5; Very exaggerated: *t*(142) = 8.9, *p* < .001, *d* = 2).

**Mapping direction** Bonferroni corrected t-tests comparing Mapping scores to 0 revealed that participants had a clear preference for Congruent mappings if movements were exaggerated, yet there was no such preference for non-exaggerated (i.e., Normal) movements (*t*(24) = -1.4, *p* = .08, *d* = .5 ) (Fig 3B)

**Fig 3. Results of Partial distal goal condition.**

Distribution of A. Consistency scores and B. Mapping scores in the Partial distal goal condition, for the three degrees of exaggeration. Each dot represents an individual participant, with one Consistency or Mapping score for each degree of exaggeration.

**Effect of rescaling on Consistency and Mapping scores** We conducted Bonferroni corrected *t*-tests to assess the effect of our rescaling procedure on participant’s Consistency scores. These tests revealed significant differences between the Proximal goal and Partial distal goal conditions across all three degrees of exaggeration (all *t*(184) > 2.56, *p* < .03, *d* > .6), while none of the tests comparing the Partial distal goal and Distal goal conditions reached significance (all *t*(186) > -.9, *p* > .16, *d* < .5).

Bonferroni corrected *t*-tests were also conducted to analyze the effect of rescaling on the Mapping scores. The tests revealed significant differences between the Partial distal goal and the Proximal goal conditions only when movements were Normal, (*t*(153) = 3.9, *p* < .001, *d* = 1.8 ), but not when they were exaggerated. None of the tests comparing the Distal goal and Partial distal goal conditions reached significance (all *t*(153) > -2.3, *p* > .06, *d* < .5).

These results suggest that participants were affected by the rescaling procedure introduced in the Partial distal goal condition, even if they were not aware of the fact that the movements converged towards a single endpoint.
